# Co-Transmission of Alpha-Synuclein and TPPP/p25 Inhibits Their Proteolytic Degradation in Human Cell Models

**DOI:** 10.3389/fmolb.2021.666026

**Published:** 2021-05-18

**Authors:** Attila Lehotzky, Judit Oláh, János Tibor Fekete, Tibor Szénási, Edit Szabó, Balázs Győrffy, György Várady, Judit Ovádi

**Affiliations:** Institute of Enzymology, Research Center for Natural Sciences, Budapest, Hungary

**Keywords:** parkinsonism, alpha-synuclein, TPPP/p25, autophagy inhibition, drug target

## Abstract

The pathological association of alpha-synuclein (SYN) and Tubulin Polymerization Promoting Protein (TPPP/p25) is a key factor in the etiology of synucleinopathies. In normal brains, the intrinsically disordered SYN and TPPP/p25 are not found together but exist separately in neurons and oligodendrocytes, respectively; in pathological states, however, they are found in both cell types due to their cell-to-cell transmission. The autophagy degradation of the accumulated/assembled SYN has been considered as a potential therapeutic target. We have shown that the hetero-association of SYN with TPPP/p25 after their uptake from the medium by human cells (which mimics cell-to-cell transmission) inhibits both their autophagy- and the ubiquitin-proteasome system-derived elimination. These results were obtained by ELISA, Western blot, FACS and immunofluorescence confocal microscopy using human recombinant proteins and living human cells; ANOVA statistical analysis confirmed that TPPP/p25 counteracts SYN degradation by hindering the autophagy maturation at the stage of LC3B-SQSTM1/p62-derived autophagosome formation and its fusion with lysosome. Recently, fragments of TPPP/p25 that bind to the interface between the two hallmark proteins have been shown to inhibit their pathological assembly. In this work, we show that the proteolytic degradation of SYN on its own is more effective than when it is complexed with TPPP/p25. The combined strategy of TPPP/p25 fragments and proteolysis may ensure prevention and/or elimination of pathological SYN assemblies.

## Introduction

Autophagy is a catabolic process that leads to the degradation of unwanted cytoplasmic components such as protein assemblies (Lamb et al., 2013). Different types of autophagy can be distinguished based on the distinct pathways by which the cargo is delivered to the lysosomes. Macroautophagy (autophagy) is the major pathway, which involves the engulfment of the cargo by a double-membrane phagophore that elongates and seals to form an autophagosome by the active involvement of the autophagy markers, microtubule-associated protein light chain 3 (LC3B) and sequestosome one denoted SQSTM1/p62. LC3B and SQSTM1/p62 are indicators of the formation of autophagosomes. LC3B-II is formed by the covalent linking of phosphatidylethanolamine to LC3B, which is incorporated into the phagophore membrane and its level tightly controls the size of autophagosomes (Lilienbaum, 2013). The newly formed autophagosome then fuses with the lysosome forming the autolysosome ensuring an acidic milieu for the pH-dependent degradation of proteins, lipids, nucleic acids, and carbohydrates (Kimura et al., 2007; Lamb et al., 2013).

The function of autophagy is to allow cells to degrade and recycle damaged organelles and proteins; it is an important pathway implicated in neuronal health and diseases (Pandey et al., 2007; Nedelsky et al., 2008; Lee et al., 2013). The degradative potencies of autophagy and the ubiquitin-proteasome system (UPS) ensure the major protein quality control mechanisms in eukaryotic cells (Lilienbaum, 2013; Nam et al., 2017). Autophagy ensures adaptive response to nutrient deprivation, as well as to the survival of neurons by counteracting their dysregulation, which leads to neurodegeneration such as synucleinopathies (Pandey et al., 2007; Nedelsky et al., 2008; Lee et al., 2013). Parkinson’s disease (PD) is one of the age-associated neurodegenerative disorders characterized by progressive neuronal cell loss and widespread aggregation of alpha-synuclein (SYN) forming Lewy bodies (Kalia and Lang, 2015; Mochizuki et al., 2018).

SYN is a disordered protein, prototype of the chameleon proteins; it is predominantly unfolded and capable of adopting structurally unrelated conformations (Uversky, 2003). It is expressed predominantly in neurons and neutrophil cells (Maroteaux et al., 1988; Bates and Zheng, 2014). The physiological function of SYN is still unclear; it seems to be involved in the modulation of neuronal plasticity, synaptic vesicle pool maintenance and dopamine metabolism (Bellucci et al., 2016; Mochizuki et al., 2018). Indeed, SYN plays multiple pivotal roles in neurodegenerative diseases; its small, soluble oligomeric forms with beta-sheet conformation are now considered the most toxic species in the etiology of PD (Ono, 2017; Surguchev and Surguchov, 2017).

The association of SYN with Tubulin Polymerization Promoting Protein (TPPP/p25), another hallmark of synucleinopathies, results in the formation of their pathological complex (Oláh and Ovádi, 2019; Oláh et al., 2020). TPPP/p25 is also a brain-specific disordered protein; although it is barely expressed, if at all, in neurons, it is expressed in oligodendrocytes (OLGs) where it is crucial for their differentiation and for the ensheathment of the axons (Lehotzky et al., 2010; Oláh et al., 2020). The tendency of TPPP/p25 to aggregate and to drive pathological SYN assembly is well-established (Oláh and Ovádi, 2019; Oláh et al., 2020). Their co-enrichments and co-localizations have been visualized in neurons as Lewy bodies and in OLGs as glial cytoplasmic inclusions in brains of PD and multiple system atrophy (MSA) patients, respectively (Kovacs et al., 2004).

The presence of both proteins in the extracellular space has been suggested by their occurrence in the cerebrospinal fluid (Vincze et al., 2011; Marques and Outeiro, 2012; Andersen et al., 2017) and by their uptake from the medium by cells (Tőkési et al., 2014; Szunyogh et al., 2015); in addition, the cell-to-cell transmission of SYN has also been reported (Danzer et al., 2012; Menendez-Gonzalez et al., 2018; Valdinocci et al., 2018; Surguchev et al., 2019). Although the mechanism of this transmission is as yet unclear, the liberation of the endocytosed materials in the cytoplasm by the mechanism of “endosomal escape” to reach autophagic vacuole has been proposed (Smith et al., 2019). This mechanism could operate in the case of the exogenously supplied SYN and/or TPPP/p25. Moreover, TPPP/p25-overexpressing oligodendroglial cells taking up human pre-formed SYN fibrils can form insoluble, highly aggregated, pathological assemblies, which leads to the disruption of the microtubule and myelin networks (Mavroeidi et al., 2019).

Previously, we reported the co-enrichment and co-localization of TPPP/p25 and SYN in mammalian cells resulting from their uptake from the medium, which led to their aggregation and mimicked the pathological situation (Lehotzky et al., 2004; Tőkési et al., 2014; Szunyogh et al., 2015; Szénási et al., 2017). Therefore, human cell models appear suitable for studying the formation of the pathological inclusions occurring in neurons and in OLGs, which are characteristic of PD and MSA, respectively. In this work, we have used human HeLa and SH-SY5Y cells, which express neither SYN nor TPPP/p25, at least not in amounts corresponding to the pathological situation. The uptake of the human recombinant SYN and/or TPPP/p25 from the medium by these cells allows evaluation of the effect of TPPP/p25 on the elimination of the accumulated intracellular SYN.

## Materials and Methods

### Chemicals and Antibodies

The source of chemicals and antibodies are listed in Table 1.

**TABLE 1:** Key chemicals and antibodies used in the study.

Chemical/Antibody	Company	Catalog number	Concentration/Dilution
MG132	Sigma-aldrich	C2211	5 μM
Temsirolimus^a^	Sigma-aldrich	PZ0020	1 μg/ml
CQ	Sigma-aldrich	C6628	5 μM
Rat polyclonal anti-TPPP/p25	Kovacs et al. (2004)		1:5,000, 1:2,000^b^
Mouse monoclonal anti-SYN	Sigma-aldrich	S5566	1:1,000^b^
Rabbit polyclonal anti-SYN	Sigma-aldrich	S3062	1:5,000
Rabbit polyclonal anti-SQSTM1/p62	Sigma-aldrich	P0067	1:5,000, 1:1,000^b^
Rabbit polyclonal anti-LC3B	Cell signaling technology	2775	1:1,000
anti-GAPDH	Sigma-aldrich	G9545	1:5,000
Anti-rat IgG, HRP-linked	Sigma-aldrich	A9037	1:5,000
Anti-mouse IgG, HRP-linked	Sigma-aldrich	A2554	1:5,000
Anti-rabbit IgG, HRP-linked	Thermo Fisher scientific	32,260	1:5,000
Anti-mouse IgG, Alexa-488 linked	Thermo Fisher scientific	A11029	1:1,000^b^
Anti-rat IgG, Alexa-488 linked	Thermo Fisher scientific	A11006	1:1,000^b^
Anti-rabbit IgG, Alexa-488 linked	Thermo Fisher scientific	A11008	1:1,000^b^
Anti-rat IgG, Alexa-546 linked	Thermo Fisher scientific	A11081	1:1,000^b^

a Temsirolimus is an esterified rapamycin.

b Dilution for immunocytochemistry.

### Plasmid Constructs

Prokaryotic expression plasmids containing the insert for human TPPP/p25 and SYN were prepared and purified as described previously, respectively (Tőkési et al., 2010; Szunyogh et al., 2015). The pET-mCherry-LC3B construct was a gift from Sascha Martens (Max Perutz Labs, University of Vienna, Austria) (Zaffagnini et al., 2018). The pmRFP-LC3B construct was a gift from Nicholas Ktistakis (Babraham Institute, Cambridge, United Kingdom) (Axe et al., 2008).

The Bimolecular Fluorescence Complementation (BiFC) plasmids (pBiFC-VN1-173, pBiFC-VC155–238) were a gift of Prof. Péter Várnai (Semmelweis University, Budapest). The V^C^-TPPP/p25 plasmid containing the insert for human full length TPPP/p25 was prepared as described previously (Oláh et al., 2017). The V^N^-LC3B was produced by inserting the LC3B coding region from pmRFP-LC3B construct in frame into pBiFC-VN1-173 by BglII and EcoRI restriction enzymes. The V^C^- SQSTM1/p62 was produced by inserting the SQSTM1/p62 coding region from EGFP-SQSTM1/p62 (Linares et al., 2013) in frame into pBiFC-VC155–238 by BglII and SalI restriction enzymes. The sequences of all construct were verified by restriction mapping and sequencing.

### Expression and Purification of Human Recombinant Proteins

Human recombinant SYN was prepared as described previously (Paik et al., 1997). Human recombinant TPPP/p25 with hexaHis tag and TPPP/p25 mutants were expressed in *E. coli* BL21 (DE3), and was isolated on HIS-Select™ Cartridge (Sigma-Aldrich) as described previously (Tőkési et al., 2014; Szénási et al., 2017).

LC3B fused with mCherry was prepared as described by (Wurzer et al., 2015). The ampicillin-resistant cells were grown in Luria Bertani Broth containing 100 mg/L ampicillin, and 1 mM MgCl_2_ at 37°C till 1.0 absorbance at 600 nm, then protein expression was induced by the addition of 0.1 mM isopropyl *β*-D-1-thiogalactopyranoside. The cells were further incubated at 18°C for 16 h, then harvested by centrifugation (20 min, 4°C, 2,000 g). The pellet fraction was resuspended in 50 mM 4-(2-hydroxyethyl)-1-piperazineethanesulfonic acid, 500 mM NaCl, pH 7.5 buffer (containing 10 mM imidazole, 2 mM MgCl_2_, 2 mM 2-mercaptoethanol, 10 μM 4-(2-aminoethyl)benzenesulfonyl fluoride hydrochloride, 1 mM benzamidine, 1 μg/ml pepstatin, 1 μg/ml leupeptin, 0.1% Triton X-100 and 10 μg/ml DNAse), and was lyzed by sonication. Following centrifugation (30 min, 4°C, 44,000 g), the supernatant containing the soluble proteins was affinity purified by Ni^2+^ affinity gel (Sigma P6611). The bound protein was eluted by 50 mM 4-(2-hydroxyethyl)-1-piperazineethanesulfonic acid, 500 mM NaCl, pH 7.5 containing 100 mM imidazole, then it was concentrated and stored in 25 mM 4-(2-hydroxyethyl)-1-piperazineethanesulfonic acid, 150 mM NaCl pH 7.5, and 1 mM dithiothreitol.

### Protein Determination

Concentrations of the purified human recombinant proteins were determined on the basis of their absorbance at 280 nm using the extinction coefficients evaluated by ExPASy-ProtParam tool (http://web.expasy.org/protparam): 5960 M^−1^*cm^−1^ for SYN, 10,095 M^−1^*cm^−1^ for TPPP/p25, 5625 M^−1^ *cm^−1^ for double truncated TPPP/p25 (Δ3-43/Δ175–219 TPPP/p25), 5625 M^−1^ *cm^−1^ for rfr-1 (43–90 TPPP/p25), 4470 M^−1^ *cm^−1^ for rfr-3 (142–219/Δ178–187 TPPP/p25) and 41,830 M^−1^*cm^−1^ for mCherry-LC3B, respectively.

The total protein concentration of cell extracts was measured by the Bradford method (Bradford, 1976) using the Bio-Rad protein assay kit.

### Enzyme-Linked Immunosorbent Assay

ELISA experiments were carried out similarly as described previously (Tőkési et al., 2014). Briefly, the plate was coated with 5 μg/ml (50 μL/well) mCherry-LC3B in phosphate buffered saline (PBS). After blocking the wells with bovine serum albumin, the immobilized protein was incubated with serial dilutions of TPPP/p25 forms or SYN; the bound TPPP/p25 forms or SYN were detected by specific TPPP/p25 (Kovacs et al., 2004) or SYN (Sigma S3062) antibodies followed by the addition of the peroxidase conjugated secondary IgGs.

For the ELISA experiment carried out with cell extracts, the cell lysates were prepared from HeLa cells grown on 60 mm cell culture dishes harvested at 80% confluence. The cells were detached by trypsin–EDTA solution, washed with medium, then three times with PBS, centrifuged (2 min, room temperature, 200 g) between the washing steps, then resuspended in lysis buffer (50 mM tris(hydroxymethyl)aminomethane, 100 mM NaCl, pH 7.5 containing 0.5% NP-40). The HeLa cell extract was dialyzed in PBS buffer for 30 min at 4°C, then centrifuged (10 min, 4°C, 17,000 g), and the resulting supernatant was used for ELISA experiments. In this experimental setup, the plate was coated with 5 μg/ml (50 μL/well) mCherry-LC3B, TPPP/p25 or SYN, and after blocking the wells with bovine serum albumin, the immobilized proteins were incubated with HeLa cell extract at various concentrations. The bound endogenous SQSTM1/p62 from the extract was detected by rabbit polyclonal SQSTM1/p62 antibody (Sigma P0067) and the corresponding peroxidase conjugated secondary IgG.

The immunocomplex was quantified using o-phenylenediamine with hydrogen peroxide as substrate solution. The reaction was stopped after 10 min with 1 M H_2_SO_4_, and the absorbance was read at 490 nm with an EnSpire Multimode Reader (Perkin Elmer). The apparent binding constants (K_d_) were evaluated from the saturation curves through non-linear curve fitting using a hyperbola model with single binding site (Origin 2018 64Bit software).

### Cell Culture, Transfection and Manipulation

HeLa cells (ATCC^®^ CCL-2™) and LC3B-HeLa (a gift from Research Institute for Microbial Diseases, Osaka University, Osaka, Japan) cells expressing tandem fluorescent-tagged LC3B (monomeric red fluorescent protein-enhanced green fluorescent protein LC3B, mRFP-EGFP-LC3B) (Kimura et al., 2007) were grown in Dulbecco’s Modified Eagle’s Medium (high glucose) supplemented with 10% (v/v) fetal bovine serum (FBS), 100 μg/ml kanamycin and Antibiotic Antimycotic Solution (all from Sigma-Aldrich; complete medium) in a humidified incubator at 37°C with 5% CO_2_.

Neuroblastoma SH-SY5Y cells (ATCC CRL-2266) were cultured in DME/F12 medium, supplemented with 10% FBS, 100 μg/ml kanamycin and Antibiotic Antimycotic Solution (all Sigma-Aldrich; complete medium). Cells were grown in a humidified incubator at 37°C, in a 5% CO_2_ atmosphere. The line was used between 10 and 30 passages for experiments.

For cellular experiments, 5 × 10^4^ HeLa, SH-SY5Y and LC3B-HeLa cells, respectively, were plated in 24-well plates. For mRFP-LC3B experiments, 100 ng plasmid was transfected with TurboFect (Thermo Fischer) transfection reagent into coverslip-plated HeLa cells according to the manufacturer’s suggestions. The human cells (HeLa or SH-SY5Y or LC3B-HeLa) were grown in low glucose and low protein medium (2 hs pre-incubation in 2% FBS/low glucose DMEM with 100 μg/ml kanamycin) before adding the small molecules or recombinant proteins, when indicated. After addition of effectors and/or protein, cells were incubated for further 4 h. The concentrations of MG132, rapamycin and chloroquine (CQ) were 5 μM, 1 μg/ml and 5 μM (Table 1); while the concentrations of TPPP/p25 and SYN were 80 and 800 nM, respectively. Mixtures of SYN and TPPP/p25 (MIX) was prepared from 1.0 mg/ml stocks solutions in a sterile tube, then added to the medium of cells.

For the detection of the BiFC signal, HeLa cells were transfected with the mVenus BiFC constructs of V^N^-LC3B, V^C^-TPPP/p25 and V^C^-SQSTM1/p62 (100–100 ng for each V^N^-V^C^ pair) using Turbofect (Invitrogen) transfection reagent according to the manufacture’s protocol. Nuclei were counterstained with DAPI. BiFC signal was detected on formaldehyde-fixed (4% in PBS) samples by confocal microscopy.

For microscopy, HeLa, SH-SY5Y or LC3B-HeLa cells on coverslips were fixed by cold methanol, then processed for routine immunocytochemistry or were detected for the fluorescent constructs directly. Concentrations of reagents (Table 1) or proteins (80 nM for TPPP/p25 and 800 nM for SYN, respectively) were the same as above.

For fluorescence-activated cell sorting (FACS) analysis, treated LC3B-HeLa cells were trypsinized from 24-wells of a plate. During trypsinization, cells were covered with PBS (100 μL), next 100 μL 2% FBS in PBS was added to the cells to block clumping in sample, then samples from wells were collected into a 96-well plate, V-shaped for FACS analysis.

For Western blot, the HeLa or SH-SY5Y cells were washed with PBS, lyzed *in situ* in 1x reducing sample buffer for sodium dodecyl sulfate polyacrylamide gel electrophoresis (SDS-PAGE) followed by Western blot.

### Western Blot Analysis

The samples were analyzed by 13.5% SDS-PAGE after loading equal amount of proteins and were electrotransferred onto Immobilon-PSQ transfer membranes and subjected to Western blot. Post-transfer membranes were treated with 2% paraformaldehyde in PBS containing 0.1% Tween-20 for 30 min and then washed three times with PBS containing 0.1% Tween-20 before blocking. The blot was developed using a rabbit polyclonal SYN antibody against the epitope of 111–132 aa (Sigma S3062), a rat polyclonal TPPP/p25 antibody (Kovacs et al., 2004), and a rabbit polyclonal SQSTM1/p62 antibody (Sigma P0067) or a rabbit polyclonal GAPDH antibody sequentially (Table 1). Antibody binding was revealed by the corresponding IgG-peroxidase conjugate. To quantify LC3B levels, the same samples were also analyzed by 16% SDS-PAGE, then electrotransferred onto Immobilon-PSQ membranes followed by Western blot. The blots were developed using a rabbit polyclonal LC3B antibody (Cell Signaling Technology, 2775).

Peroxidase reaction was detected using Immobilon Western substrate (Millipore) by a Bio-Rad ChemiDoc MP Imaging system and its ImageLab 4.1 software. Then amido black solution (0.1% w/v amido black, 25% v/v isopropanol and 10% v/v acetic acid) was applied to stain the protein bands on the membrane. Intensity of bands was analyzed by ImageJ 1.49 using Measure command. The densitometric analysis was performed as described (Butler et al., 2019). Briefly, the region of interest encircling each band was defined manually. All bands at the correct molecular weight ± approximately 5 kDa were analyzed as the signal for the given protein. In this region, any overlapping visible bands in the image were included to ensure that the level of background signal subtraction was appropriate to the level of background noise. Data normalization was performed by dividing the value of the target protein by the value of the chosen loading control (GAPDH) in the case of SH-SY5Y cells.

### Fluorescence-Activated Cell Sorting

The cell line expressing tandem fluorescent-tagged LC3B (LC3B-HeLa) was measured by Attune NxT Acoustic Flow cytometer (Invitrogen by Thermo Fisher Scientific) equipped with four lasers and Autosampler. The EGFP fluorescence was detected using blue laser excitation (488 nm) and 530/30 nm emission (FL1 channel). The mRFP fluorescence was measured using yellow-green laser excitation (561 nm) and 585/16 nm emission (YL1 channel). The shift of red and green fluorescence ratio was determined in percentage and compared among the treated and untreated cell populations (Supplementary Figure S1).

### Immunofluorescence Microscopy

Confocal images were acquired with a Zeiss LSM 710 microscope using an oiled 40 × NA = 1.4 Plan Apo objective. The equipment and acquisition were controlled by the LSM Zen 2010 B SP1 software. For the excitation of fluorophores, the following lasers were used: Diode laser at 405 nm for 4′,6-diamidino-2-phenylindole (DAPI), Argon laser at 488 nm for EGFP or Alexa 488, Argon laser at 514 nm for mVenus, HeNe laser at 543 nm for mRFP or Alexa 546. Detection range of fluorophores was determined by the software using built-in emission parameters considering the fluorophores; signals were acquisited one by one.

The original pictures (2048 × 2048 pixels, 72 dpi, 8-bit lsm images) were converted to tiff format. Complex images were created with Adobe Photoshop 2020 CC. Relevant parts from the tiff images were cut, and a minimal background correction was applied. In the presented images, original pixel density was changed to at least 300 dpi. 2.0x digital zoom provided by the Zen software was used on some samples during acquisition for the aim of more detailed pictures. The number of punctas per cell was counted for each sample by an observer blinded to experimental conditions.

### Statistical Analysis

All values are presented as the mean ± SD (standard deviation) of at least 3 independent experiments. Statistical comparisons were performed with one-way or two-way ANOVA followed by Tukey’s test for multiple comparisons among control and treatments. Statistical significance was considered at *p* < 0.05. GraphPad Prism 8.3.0 (GraphPad Software, La Jolla, CA, United States) was used for all statistical analyses.

## Results

### Interactions Between the Markers of Autophagy and the Hallmark Proteins of Parkinson’s Disease

The direct interaction between the autophagy marker proteins LC3B and SQSTM1/p62 is important for the degradation of toxic protein assemblies by the basal constitutive autophagy (Pankiv et al., 2007). Most of the proteins degraded by selective autophagy can be recognized by the presence of short, conserved sequence motifs known as LC3-interacting regions (Birgisdottir et al., 2013; Xie et al., 2016; Jacomin et al., 2017). LC3-interacting region (LIR) motif (W/F/YxxI/L/V) can be found within the sequence of human TPPP/p25 (^76^WSKL^79^ and ^100^FSKI^103^), but not in that of SYN, which motivated us to test their interacting potency with LC3B.

**FIGURE 1:**
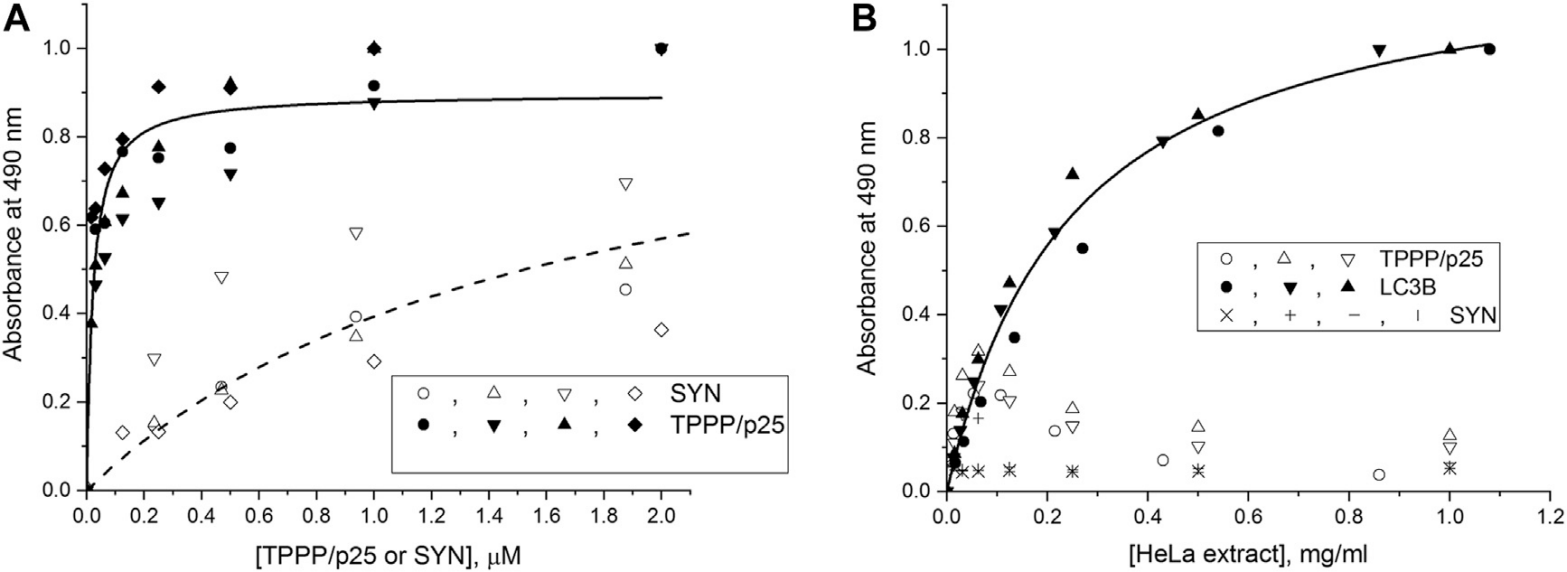
Interaction of the autophagy markers LC3B and SQSTM1/p62 with SYN or TPPP/p25 as detected by ELISA. **(A)** Binding of human recombinant TPPP/p25 (●,▲, ▼, ♦) and SYN (○, ∆, ∇, ◊) to LC3B. TPPP/p25 or SYN at different concentrations were added to LC3B immobilized on the plate, and the binding affinities evaluated by curve fitting, assuming simple hyperbolic saturation, are as follows: K_d_ = 21.5 ± 4.1 nM (TPPP/p25, *n* = 4) and 1.62 ± 0.32 μM (SYN, *n* = 4). **(B)** Binding of endogenous SQSTM1/p62 from HeLa cell extract to the immobilized LC3B (●, ▲, ▼), TPPP/p25 (○, ∆, ∇) or SYN (x, +, ǀ, —). The plate was coated with LC3B, TPPP/p25 or SYN, and the immobilized proteins were incubated with HeLa cell extract expressing endogenous SQSTM1/p62 at various concentrations. EC_50_ value evaluated by curve fitting, assuming simple hyperbolic saturation, is: EC_50_ = 0.246 ± 0.024 mg/ml for LC3B. *n* = 3 for TPPP/p25 and LC3B, *n* = 4 for SYN.

The interactions of the hallmark proteins with LC3B were studied by ELISA; TPPP/p25 or SYN were added separately at various concentrations to the LC3B immobilized on the plate, and their associations were detected by specific antibodies as described in the Materials and Methods. As shown in Figure 1A, TPPP/p25 tightly binds to the immobilized LC3B as evaluated by curve fitting (K_d_ = 21.5 ± 4.1 nM); while SYN shows much less affinity. The binding affinity of TPPP/p25 to the immobilized LC3B in the absence and presence of cell-free extracts is comparable (Supplementary Figure S2A). In addition, our data show that the interactions of LC3B with TPPP/p25 or TPPP/p25 complexed with SYN are achieved independently of the presence of the HeLa cell-free extract (Supplementary Figure S2B). To obtain information about the involvement of the LIR motifs of TPPP/p25, the binding of different deletion mutants of TPPP/p25 to LC3B was tested. Supplementary Figure S2C shows that the binding affinity of the CORE segment of TPPP/p25 (Δ3-43/Δ175–219 TPPP/p25), containing both LIR motifs, is comparable with that of the wild type protein; while the binding of the TPPP/p25 mutants, such as rfr-1 (Δ43–90) or rfr-3 (Δ142–219/Δ178–187), to LC3B is weaker or much less, if at all. These results suggest the involvement of LIR motif(s) in the hetero-association of LC3B with TPPP/p25.

Direct evidence for the intracellular association of LC3B with TPPP/p25 was obtained by fluorescence confocal microscopy coupled with BiFC technology. mVenus vectors (V^N^-LC3B and V^C^-TPPP/p25 as well as V^N^-LC3B and V^C^-SQSTM1/p62) were used to visualize the binding of TPPP/p25 to LC3B. The N-terminal and the C-terminal domains of a fluorescent protein (Venus) are fused separately to the two partner proteins, their intracellular hetero-association brings the two domains of the split fluorescent Venus protein in close proximity, which results in a fluoresce emission upon excitation, a BiFC signal. Supplementary Figure S3 illustrates the hetero-association of TPPP/p25 and LC3B in HeLa cells, as a positive control, the association of LC3B and SQSTM1/p62 is shown (Supplementary Figure S3).

Next, TPPP/p25 or SYN was immobilized on the plate, and then the extract of human cells expressing endogenous SQSTM1/p62 was added at various concentrations, and the bound SQSTM1/p62 was quantified by a specific SQSTM1/p62 antibody. As a positive control, LC3B, which interacts with SQSTM1/p62, was immobilized on the plate instead of TPPP/p25 or SYN. As shown in Figure 1B, neither TPPP/p25 nor SYN are associated with SQSTM1/p62, while the immobilized LC3B did, as expected, bind the endogenous SQSTM1/p62 (Pankiv et al., 2007; Novak et al., 2010). These findings showed the potential influence of TPPP/p25 on the degradation of protein assemblies via its association with the autophagy marker LC3B but not with SQSTM1/p62.

Next, the effects of the hallmark proteins, SYN and TPPP/p25, on the association between LC3B and SQSTM1/p62 were studied in a living human cell model. The co-localization of mRFP-tagged LC3B (red) expressed by transient transfection with the endogenous SQSTM1/p62 stained using specific antibody (green) was visualized by immunofluorescence confocal microscopy.

**FIGURE 2:**
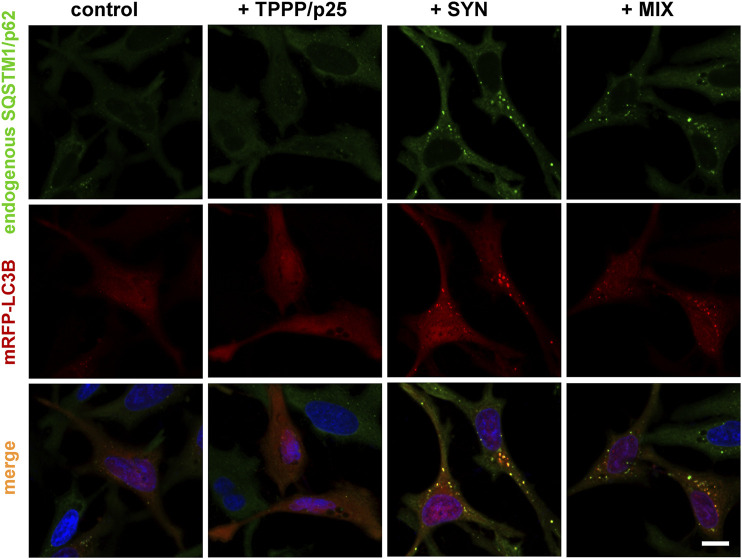
Representative images illustrate the effects of TPPP/p25 and/or SYN on the co-localization of LC3B (red) and endogenous SQSTM1/p62 (green). SYN, TPPP/p25 or their mixture (MIX, 80 nM TPPP/p25 and 800 nM SYN) were added to the medium of HeLa cells transfected with mRFP-LC3B as described in the Materials and Methods; and the co-localization of the autophagosome markers was visualized by immunofluorescence confocal microscopy; nuclei were counterstained with DAPI (blue). Scale bar: 5 μm.

**FIGURE 3:**
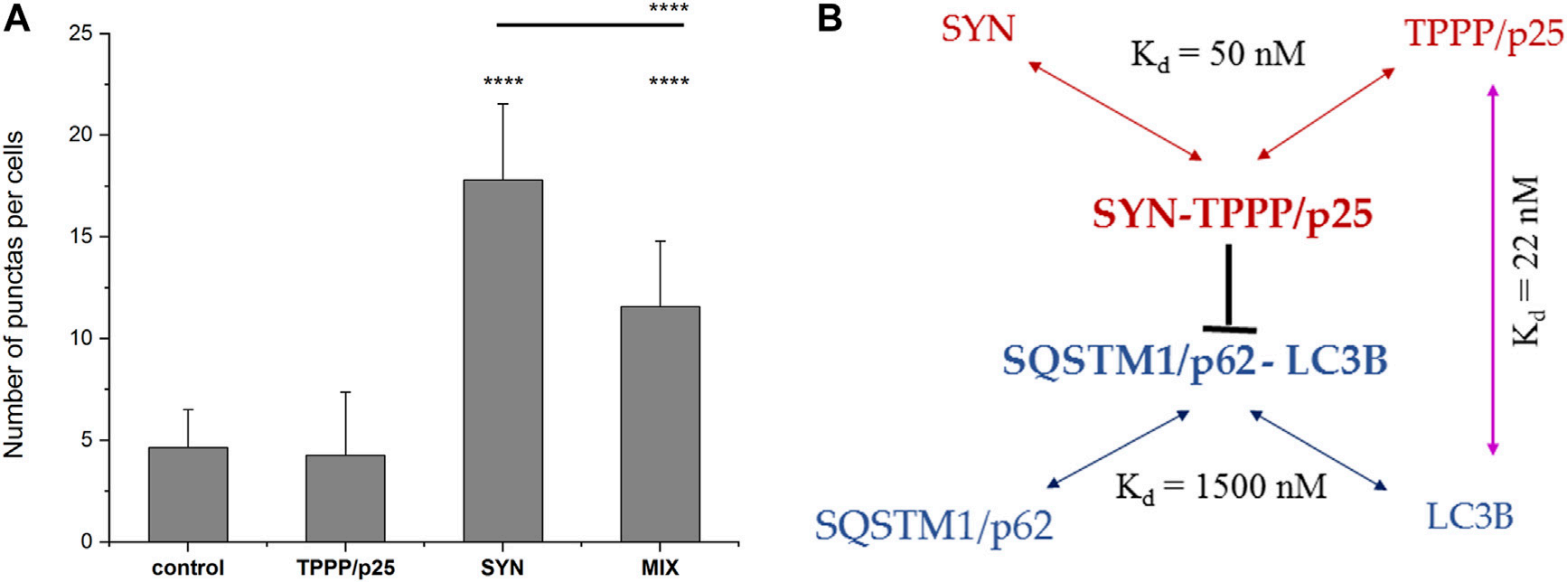
Interactions of the markers of autophagy and the hallmarks of PD. **(A)** Quantification of the effects of SYN and/or TPPP/p25 on the co-localization of LC3B (mRFP-tagged LC3B expressed by transient transfection) and the endogenous SQSTM1/p62 (yellow puncta) (as shown by a representative image in Figure 2). Statistical comparisons were performed with one-way ANOVA followed by Tukey’s test. Number of punctas per cells: ****p < 0.0001 for control vs. SYN or MIX, and SYN vs. MIX. **(B)** The results obtained for the mutual interactions of the autophagy markers and the hallmark proteins are summarized in the scheme. K_d_ = 1500 nM for the interaction of SQSTM1/p62 and LC3B (Novak et al., 2010).

As shown in Figure 2, SYN, but not TPPP/p25, induces the co-localization of the two autophagy markers as indicated by the appearance of puncta (yellow), consistent with SYN promoting the assembly of LC3B and SQSTM1/p62. This action of SYN is reduced when TPPP/p25 was added to SYN (MIX), as supported by ANOVA statistical analysis (Figure 3). Autophagosome formation driven by SYN is reduced when TPPP/p25 is present, likely due to the direct binding of TPPP/p25 to LC3B and/or SYN (Figures 1A, 3B).

### Concentration-dependent Assembly of Alpha-Synuclein With TPPP/p25

The concentration-dependent, intracellular interaction of SYN and TPPP/p25 was established in living human cells, which express SYN and TPPP/p25 endogenously at low levels, if at all, corresponding to the wild-type state. SYN and TPPP/p25 co-exist exclusively in pathological conditions due to their cell-to-cell transmission via the extracellular space (Kovacs et al., 2004; Oláh et al., 2020). The uptake of TPPP/p25 from the medium (and not its ectopic expression) was also necessitated by the fact that the endogenously expressed TPPP/p25 occurring in OLGs associates with the microtubule network that counteracts the formation of its pathological assemblies with SYN (Tőkési et al., 2014; Szunyogh et al., 2015; Szénási et al., 2017). The mutual enrichments of SYN and TPPP/p25, corresponding to the pathological situation, were carried out by their uptake from the medium as established previously (Tőkési et al., 2014; Szunyogh et al., 2015; Szénási et al., 2017).

**FIGURE 4:**
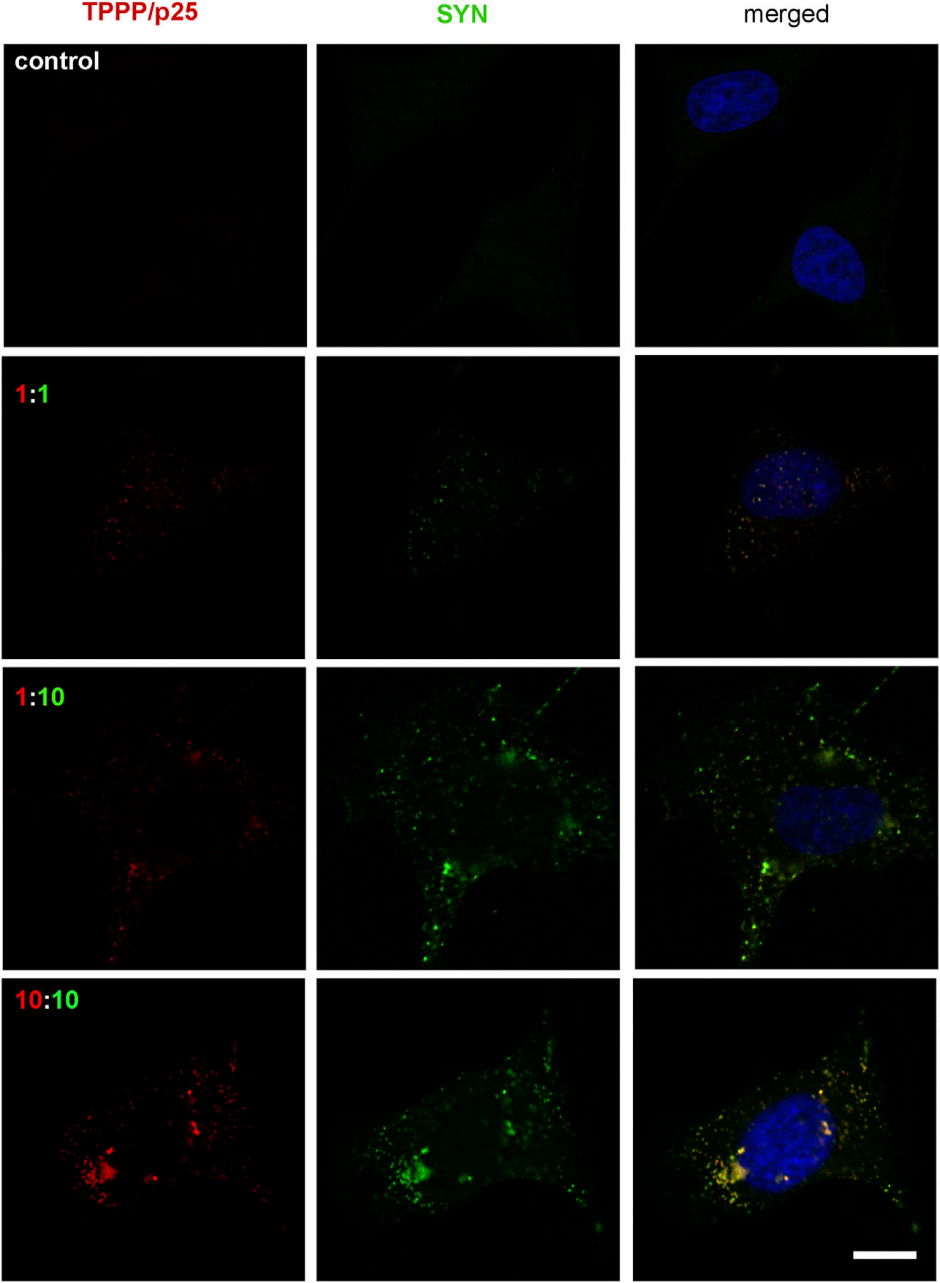
Visualization of the assembly of SYN (green) and TPPP/p25 (red) by fluorescence confocal microscopy at different ratios and concentrations of proteins in pre-starved HeLa cells. Proteins were added to the medium of cells, incubation time was 4 h. The 1:1 equimolar concentration corresponds to 80 nM. Representative images were evaluated by specific TPPP/p25 and SYN antibodies. Nuclei were counterstained with DAPI (blue). Scale bar: 5 μm.

Different concentrations and ratios of the two hallmark proteins were used to set up a cell model for the autophagy studies. At a ten-fold molar excess of SYN (TPPP/p25:SYN 80:800 nM), the co-localization of the two hallmark proteins was clearly shown by immunofluorescence confocal microscopy (Figure 4) in agreement with the reported data that substoichiometric TPPP/p25 can promote SYN assembly (Tőkési et al., 2014; Szunyogh et al., 2015). This condition was then used to investigate the effect of TPPP/p25 on the autophagy degradation of the accumulated SYN.

### Effect of Alpha-Synuclein and/or TPPP/p25 on Autophagy Flux

One of the best ways to investigate the autophagy flux and autophagy puncta is the tandem fluorescent-tagged LC3B (mRFP-EGFP) assay, which can show accumulation of LC3B in the non-acidic autophagosome and its fusion with acidic lysosome (Kimura et al., 2007; Mizushima et al., 2010). The fluorescence of EGFP is quenched in acidic compartments, whereas that of mRFP is relatively stable even within lysosomes.

In this set of fluorescence microscopy experiments, living human cells expressing tandem fluorescent-tagged LC3B (mRFP-EGFP) were used to visualize fluorescently labeled LC3B puncta as an indicator of the formation of autophagosomes (yellow) as well as the autolysosomes (red) in the course of the maturation of autophagy (Kimura et al., 2007; Yoshii and Mizushima, 2017). In the control cells, the basic autophagy proceeds at a low level (red puncta); CQ, as an inhibitor of the fusion of autophagosomes with lysosomes (Pasquier, 2016), enhances the accumulation of the autophagosomes (yellow puncta), while rapamycin, a potent inducer of autophagy (Ravikumar et al., 2004), promotes the formation of autolysosomes (red) indicating the activation of autophagy (Supplementary Figure S4). This methodology was used to establish the effects of the hallmark proteins on autophagy maturation by monitoring the LC3B behavior of the cells expressing tandem fluorescent-tagged LC3B (mRFP-EGFP) in the absence and presence of SYN and/or TPPP/p25 by fluorescence confocal microscopy.

**FIGURE 5:**
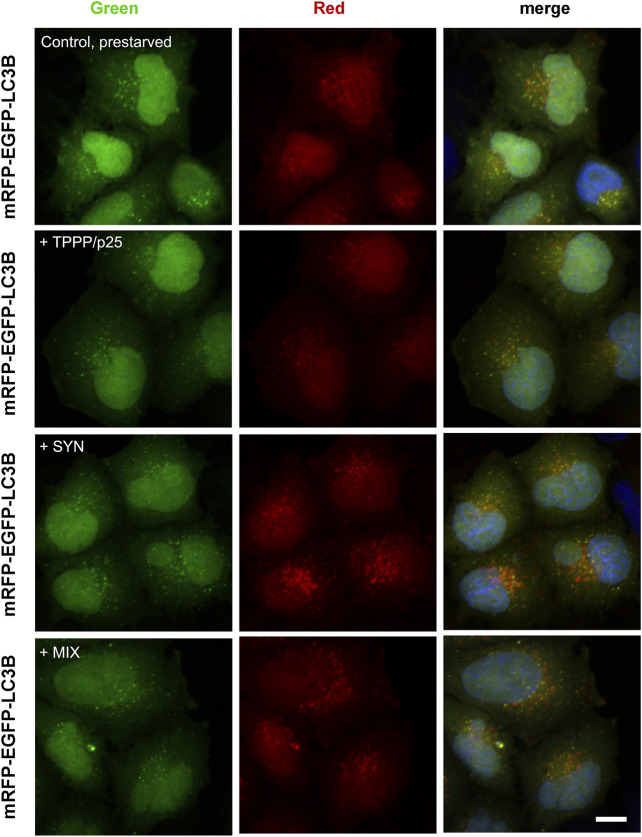
Effects of SYN and/or TPPP/p25 on the autophagy maturation in LC3B-HeLa cells expressing tandem fluorescent-tagged LC3B (mRFP-EGFP) as detected by fluorescence confocal microscopy. Pre-starved cells took up TPPP/p25 (80 nM), SYN (800 nM) or their mixture (MIX, 80 nM TPPP/p25 and 800 nM SYN) from the medium. Fluorescently labeled LC3B puncta serve as an indicator of the formation of autophagosomes (yellow) as well as the autolysosomes (red). Nuclei were counterstained with DAPI (blue). Scale bar: 2.5 μm.

**FIGURE 6:**
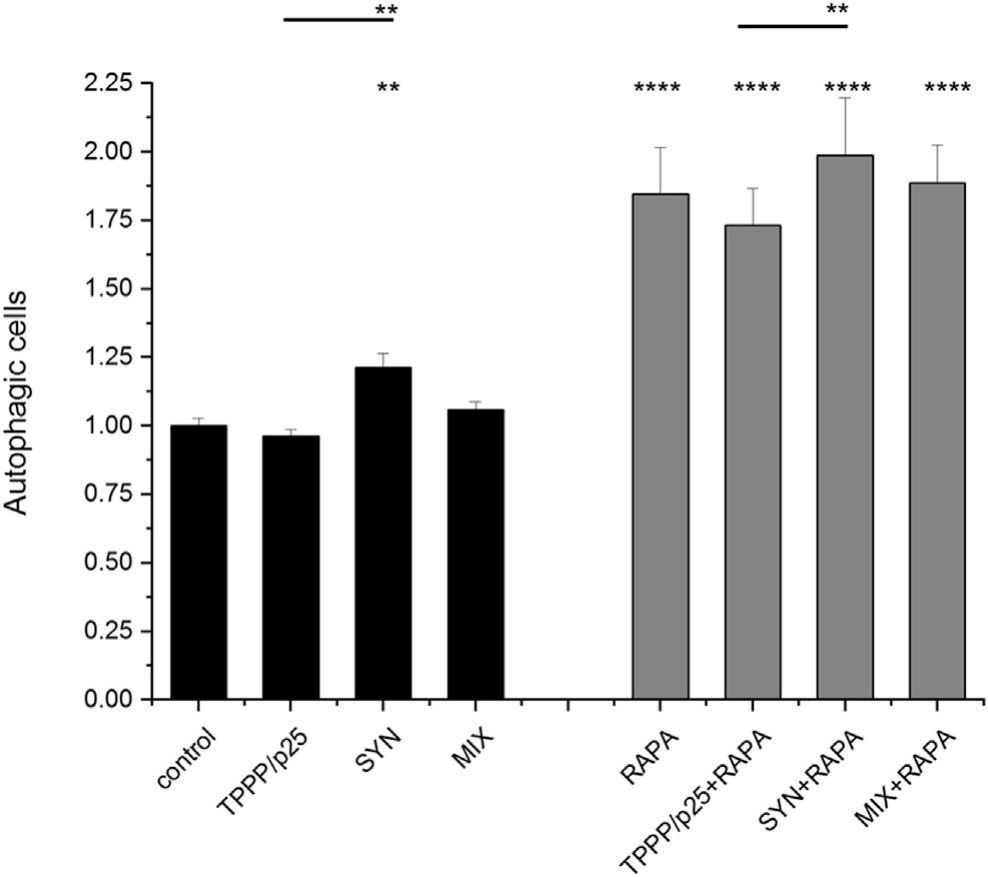
Influence of SYN and/or TPPP/p25 on the autophagy flux in the LC3B expressing-HeLa cells as detected by FACS. The concentrations of TPPP/p25 and SYN were 80 nM and 800 nM, respectively; the concentration of rapamycin (RAPA) is 1 μg/ml. Data are normalized with respect to control (no autophagy modulators) and presented as the mean ± SD of at least 4 independent experiments. Statistical comparisons were performed with two-way ANOVA followed by Tukey’s test, *p < 0.05, **p < 0.01 and ****p < 0.0001 as compared to control or as the lines indicate).

A representative image in Figure 5 illustrates that the addition of SYN, but not TPPP/p25, enhances the maturation of the autophagy (red puncta) as compared to the control, while the addition of SYN with TPPP/p25 (MIX) seems to display a distinctly different effect. The effect of TPPP/p25 on the autophagy flux was further studied and quantified by FACS (Figure 6).

FACS was used to quantify the autophagy flux in living cells expressing tandem fluorescent-tagged LC3B (mRFP-EGFP). FACS measures the fusion of the autophagosome with the lysosomes thereby providing an acidic milieu for the proteolytic degradation of proteins included into the autolysosomes as indicated by the elimination of the acid-sensitive EGFP protein (as described above). The capacity of rapamycin to promote lysosomal fusion with the autophagosome was also tested (Mizushima et al., 2010).

As shown in Figure 6, following its uptake, SYN promoted the autophagy flux, while TPPP/p25 alone and SYN with TPPP/p25 (MIX) had no significant effect on the flux. This finding indicates that TPPP/p25 counteracts the promotion by SYN of the autophagy flux. Autophagy maturation was stimulated by rapamycin, a potent inducer of autophagy, as expected. The distinct effects of TPPP/p25 and SYN on autophagy were maintained in the presence of rapamycin, however, the effect of SYN on the autophagy on its own and in the presence of TPPP/p25 (MIX) does not differ significantly (Figure 6; Supplementary Figure S1).

### Quantification of the Degradation-Resistant Alpha-Synuclein Levels Under Different Conditions

The FACS analysis provides information on the autophagy flux, the formation of autolysosomes (acidic environment) necessary for autophagy degradation. Our additional objective was to quantify directly the hallmark proteins that were not degraded. Western blot analysis provided information on the levels of the proteolysis-resistant proteins. The hallmark protein amounts were determined in control, inhibited and activated conditions as described in the Materials and Methods. The influences of CQ, rapamycin as well as that of MG132, a specific proteasome inhibitor (Lee and Goldberg, 1998) on the SYN and/or TPPP/p25 levels were quantified.

First, the effects of the inhibitors and activator of the autophagy on the intracellular levels of LC3B-II and SQSTM1/p62 were determined. As shown in Supplementary Figures S5, S6; Supplementary Table S1, in the control experiments without added hallmark proteins, significant alterations were detected, as expected (Mizushima et al., 2010; Jiang and Mizushima, 2015): both the SQSTM1/p62 and the LC3B-II levels were increased by CQ, indicating the inhibition of the autophagy maturation, while the SQSTM1/p62 level was decreased by rapamycin. The changes caused by the compounds were small but statistically significant. From these data (Supplementary Table S1), the relative levels of LC3B-II were (starved cells + CQ)–(control starved cells) > (normal cells + CQ)–(normal cells). This result shows that this model system produces the expected significant changes in autophagy maturation by either small molecular modifiers or starvation (Jiang and Mizushima 2015), and therefore validates its use in studying the influence of TPPP/p25 on the proteolytic degradation of SYN.

**FIGURE 7:**
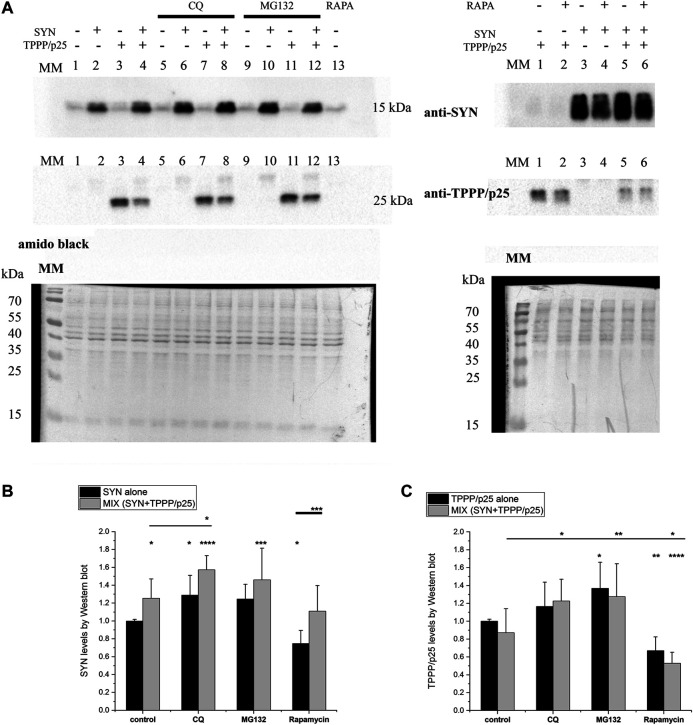
Level of the degradation-resistant hallmark proteins in the course of autophagy. TPPP/p25 and/or SYN (at 80 nM and 800 nM concentrations, respectively) were added to medium of the HeLa cells. The concentrations of CQ, rapamycin (RAPA) and MG132 are given in the Materials and Methods (Table 1). **(A)** Representative Western blots for quantification of the SYN and/or TPPP/p25 amounts detected in the absence and presence of autophagy modulators. MM: molecular weight marker. Amido black solution was applied to stain the protein bands (total protein). **(B, C)** Proteins levels of the degradation-resistant SYN **(B)** and TPPP/p25 **(C)** and their assemblies are quantified in the absence and presence of autophagy modulators by Western blot. Data are normalized with respect to the added hallmark protein alone (B: SYN, C: TPPP/p25), and presented as the mean ± SD of at least 4 independent experiments. Statistical comparisons were performed with one-way ANOVA followed by Tukey’s test (*p < 0.05, **p < 0.01, ***p < 0.001, and ****p < 0.0001 as compared to control or as the lines indicate).

As illustrated in Figure 7; Supplementary Figure S5, the relative levels of both SYN and TPPP/p25 alone increased in the presence of CQ and MG132, but decreased in the presence of rapamycin suggesting their partial elimination by both proteolytic degradative pathways. The degradation of SYN by autophagy and UPS has been reported (Ciechanover and Kwon, 2015; Stefanis et al., 2019). The increased level of TPPP/p25 in the presence of MG132 has also been reported (Lehotzky et al., 2004; Lehotzky et al., 2015). Our data add to these reports by showing that excess TPPP/p25 can also be eliminated by autophagy degradation.

Similar sets of experiments were performed in the presence of both hallmark proteins. Remarkably, as shown in Figure 7, the SYN level increased in the presence of TPPP/p25 (MIX) as compared to control (SYN alone) (cf. Figure 7B). The TPPP/p25 level does not differ significantly when the cells have taken up the protein alone (TPPP/p25) or pre-mixed with SYN (MIX) (cf. Figure 7C). These observations are supported by the statistical (ANOVA) analysis (cf. Figure 7). Further quantification of SYN and TPPP/p25 levels revealed that the mutual presence of the hallmarks does not influence the modulating effects of the inhibitors/activators significantly. The rapamycin-induced autophagy degradation is less effective, in the case of SYN assembled with TPPP/p25 (MIX) than in the case of SYN alone, while no significant difference was detected between the rapamycin samples treated with TPPP/p25 or with the MIX (Figure 7).

### Comparative Studies With SH-SY5Y Cells

Comparative experiments were carried out with human neuroblastoma SH-SY5Y cells similar to those presented above with human HeLa cells. SH-SY5Y cells can display pathological features characteristic for PD concerning at least SYN expression in neurons. The over-expression of wild-type or familial mutants of SYN has been frequently used in PD research; nevertheless, such over-expression does not always lead to increased inclusion formation. The different outcomes could be due to the specific SYN mutation used or to the expression levels achieved by the various constructs. However, an enhanced sensitivity of SH-SY5Y cells to the extracellular SYN-induced toxicity has been shown (Emadi et al., 2009). In addition, the link between SYN aggregation and other intra- and extracellular influences (abnormal mitochondrial function, oxidative stress and autophagy or proteasomal dysfunction) has been reported (Xicoy et al., 2017 and references therein).

**FIGURE 8:**
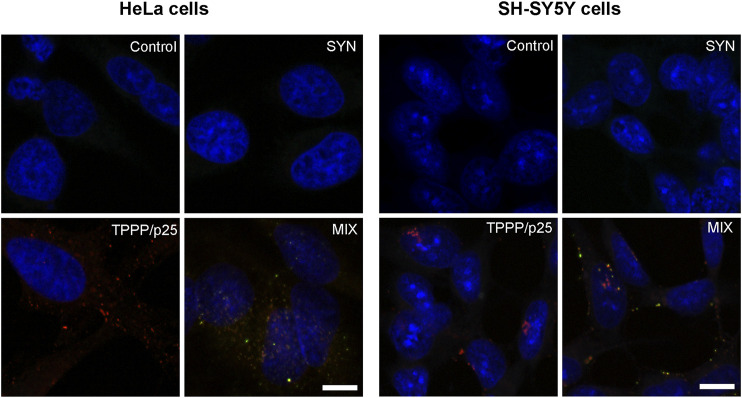
Visualization of the assembly of SYN (green) and TPPP/p25 (red) by fluorescence confocal microscopy in HeLa and SH-SY5Y cells. TPPP/p25 and/or SYN were added to the medium for their transmission into the cells. Representative images were evaluated by specific TPPP/p25 and SYN antibodies. Nuclei were counterstained with DAPI (blue). Scale bars: 10 μm.

SH-SY5Y cells do indeed express SYN at a low level that is well below the pathological level. The pathological level may be the result of uptake from the medium or from the extracellular space in living cells. Two key sets of experiments were performed simultaneously with SH-SY5Y cells and with HeLa cells. As illustrated in Figure 8, both SYN and TPPP/p25 proteins were taken up by both cell types. The assemblies of TPPP/p25 and SYN at 1:10 TPPP/p25:SYN molar ratio can be well-visualized by fluorescent microscopy (yellow puncta) when both SYN and TPPP/p25 (MIX), and not the single proteins were added to the medium.

**FIGURE 9:**
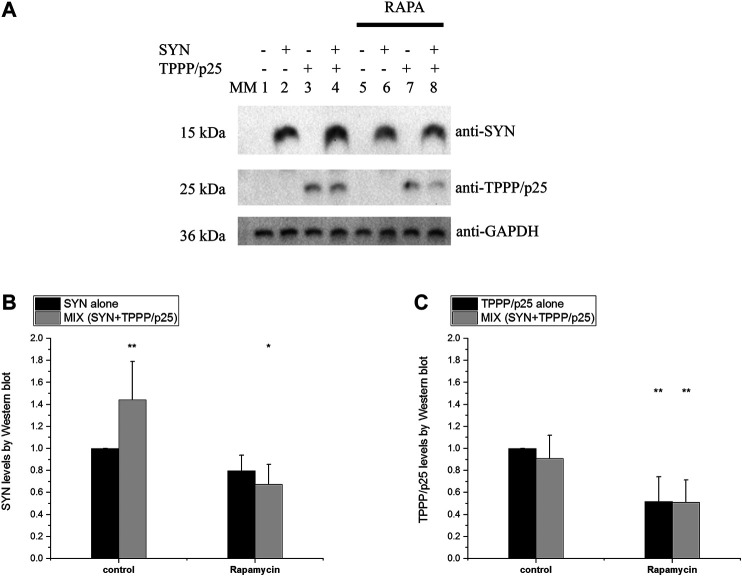
Comparative studies: level of the degradation-resistant hallmark proteins in the course of autophagy in HeLa and SH-SY5Y cells. TPPP/p25 and/or SYN at 80 nM and 800 nM concentrations, respectively, were added to the medium of SH-SY5Y cells. Other conditions are the same as described in the legend of Figure 7. The concentration of rapamycin (RAPA) is 1 μg/ml. **(A)** Representative Western blots. MM: molecular weight marker. **(B–C)** Quantification of the degradation-resistant SYN **(B)** and TPPP/p25 **(C)** and their assemblies in cells. Data are normalized with respect to the added hallmark protein alone (B: SYN, C: TPPP/p25), and presented as the mean ± SD of at least 4 independent experiments. Statistical comparisons were performed with one-way ANOVA followed by Tukey’s test (*p < 0.05 and **p < 0.01 as compared to control).

The influence of TPPP/p25 on the degradation-resistant SYN levels, as quantified by Western blotting, was similar in both cell types (Figure 9). The comparative data revealed that TPPP/p25 inhibits the proteolytic degradation of SYN: significantly higher levels of SYN (i.e., less degradation) but not of TPPP/p25, were detected in the MIX sample as compared to the proteins alone. In the course of the activation of autophagy by rapamycin, the levels of both SYN and TPPP/p25 were reduced (i.e., more degradation) as compared to the controls. In addition, in a comparative study the association of TPPP/p25 to LC3B was investigated in the presence of HeLa and SH-SY5Y cell-free extracts, and similar association was detected. All these observations suggest that the influence of TPPP/p25 on the proteolytic degradations of the pathological SYN is similar in the human cell types used in our studies.

## Discussion

PD is characterized by the loss of the dopaminergic neurons from the substance nigra (Dexter et al., 2013). Although the primary cultures of neurons from this brain area of patients seem to be the most attractive models for understanding the pathomechanism of PD, the inaccessibility and lack of proliferation of such neurons largely preclude their use (Xicoy et al., 2017). Thus, the majority of studies use a proliferative and more uniform model like the neuroblastoma SH-SY5Y cell model despite its limitations (Xicoy et al., 2017). Consequently, in many studies other cell types have been employed in parallel including a few that are not neuronal cell lines such as HeLa. The major reason for the use of HeLa cells in this work was to use a model in which the cells express endogenously low levels of SYN and TPPP/p25, if at all. Thus, the uptake of the hallmark proteins from the medium (extracellular space), their co-enrichment and co-localization can mimic situations characteristic of the synucleinopathies which generate inclusion formations in both neurons (PD) and oligodendrocytes (MSA).

In this work, different cell types and methods were used to investigate pathological SYN levels and assemblies as influenced by TPPP/p25 via proteolytic degradation/elimination. The association of LC3B and SQSTM1/p62 has a big impact on autophagy maturation at the early as well as at the later stages (Mizushima et al., 2010). Our studies suggest that the assembly of SYN with TPPP/p25 (MIX) influences the autophagy-derived proteolytic degradation of SYN involving the binding of TPPP/p25 to LC3B which inhibits the association of the autophagy markers. The Western blot analysis provided quantitative data for the non-degraded (resistant) proteins. Moreover, MG132, an inhibitor of UPS, increased the levels of TPPP/p25 and its complex with SYN (cf. Figure 7). All these data confirm that TPPP/p25 can counteract the proteolytic degradation of SYN.

The findings presented here focus on the intracellular processes occurring under pathological conditions in neurons and OLGs. The release of SYN from neurons and its transmission between neuronal and other cell types are known to drive the neurodegenerative progression called prion-like spreading (Danzer et al., 2012; Henderson et al., 2019; Fouka et al., 2020); however, the intercellular transport of TPPP/p25 is less well understood. Our studies have shown that both types of human cells can uptake SYN and/or TPPP/p25 from the medium (Tőkési et al., 2014; Szunyogh et al., 2015 and present studies). In addition, the levels of SYN are believed to be different in the cerebrospinal fluids of patients with PD or MSA (Andersen et al., 2017; Maass et al., 2019; Cong et al., 2020), while TPPP/p25 has been detected in cerebrospinal fluid (Vincze et al., 2011).

Autophagy is an evolutionarily conserved pathway (Kimura et al., 2007; Lamb et al., 2013) that is essential for neuronal survival since its dysregulation is coupled with the development of neurological disorders such as PD and MSA (Pandey et al., 2007; Nedelsky et al., 2008; Lee et al., 2013). Indeed, genetic studies have revealed extensive links between autophagy and neurodegenerative diseases, and disruptions to autophagy may contribute to the development of these diseases. Since both reduced and excessive autophagy can be detrimental, the modulation of autophagy has been proposed as a new, effective way for treating some types of neurodegenerative diseases (Mputhia et al., 2019; Parekh et al., 2019; Djajadikerta et al., 2020). Rapamycin, an allosteric mTOR inhibitor, is one of the most frequently studied autophagy inducers (Bove et al., 2011); it acts by increasing lysosomal biogenesis by avoiding autophagosome accumulation and neuronal toxicity (Dehay et al., 2010). A number of mTOR-dependent and independent autophagy inducers have been identified and some of them display beneficial effects in cellular and animal models of PD.

The destruction as well as the inhibition of the assembly of SYN and TPPP/p25 could be achieved by adopting an innovative strategy proposed recently (Tőkési et al., 2014; Szunyogh et al., 2015; Szénási et al., 2017). This strategy is based on the prevention/destruction of the pathological assemblies/inclusions by targeting the interface of the pathological SYN-TPPP/p25 complex (Tőkési et al., 2014; Szunyogh et al., 2015; Szénási et al., 2017). Experiments carried out at molecular and cellular levels have shown the importance of blocking this interface by, for example, fragments of the partner proteins depending on the type of synucleinopathy (Oláh et al., 2021). The use of drug-like agents such as peptidomimetic foldamers has also been proposed (Oláh and Ovádi, 2019; Oláh et al., 2020), although the effect of the fragments identified so far appears to be limited (Tőkési et al., 2014; Szunyogh et al., 2015; Szénási et al., 2017). Therefore, an ambitious aim would be to develop more powerful drug-like agents (competitive inhibitors) that would prevent the inhibitory effect of TPPP/p25 on the autophagic degradation of the pathological SYN. In this way, the uncomplexed SYN could perform its physiological functions, while the excess SYN could be proteolytically degraded.

## Data Availability

The original contributions presented in the study are included in the article/Supplementary Material, further inquiries can be directed to the corresponding author.

## Abbreviations

alpha-synuclein, SYN; bimolecular fluorescence complementation, BiFC; chloroquine, CQ; 4′,6-diamidino-2-phenylindole, DAPI; enhanced green fluorescent protein, EGFP; enzyme-linked immunosorbent assay, ELISA; fetal bovine serum, FBS; fluorescence-activated cell sorting, FACS; LC3-interacting region, LIR; microtubule-associated protein light chain 3, LC3B; monomeric red fluorescent protein, mRFP; multiple system atrophy, MSA; oligodendrocyte, OLG; Parkinson’s disease, PD; phosphate buffered saline, PBS; sequestosome 1, SQSTM1/p62; sodium dodecyl sulfate polyacrylamide gel electrophoresis, SDS-PAGE; Tubulin Polymerization Promoting Protein, TPPP/p25; ubiquitin-proteasome system, UPS.
