# Supplementary material for: Co-Transmission of Alpha-Synuclein and TPPP/p25 Inhibits Their Proteolytic Degradation in Human Cell Models
Source: Front Mol Biosci. 2021 May 18;8:666026. doi: 10.3389/fmolb.2021.666026 (PMC8167055; doi:10.3389/fmolb.2021.666026)
Supplement: Supplementary file 1 [file DataSheet2.pdf]

Supplementary Material

1 Supplementary Data

All primary data can be found in the Data_for_figures.xlsx Excel file.

2 Supplementary Figures and Tables

2.1 Supplementary Figures

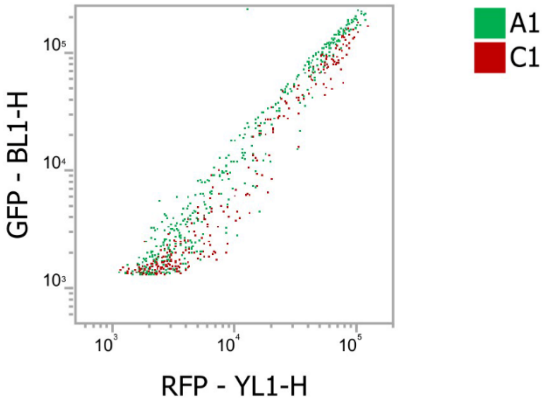

Supplementary Figure 1. Overlay dot plots of control (green dots) and rapamycin treated (red dots) pre-starved LC3B-HeLa cells as detected in the FACS experiments. The shift of the fluorescent signals due to the rapamycin treatment underlines the autophagic activation, including the percentage of autophagic cells in total cellular population (20% and 35% for the control and rapamycin-treated cells, respectively). The representative green mean fluorescence values are 38650 RFU and 28604 RFU for control and rapamycin-treated cells, while the representative red mean fluorescence values are 27271 RFU and 25401 RFU for control and rapamycin-treated cells, respectively.

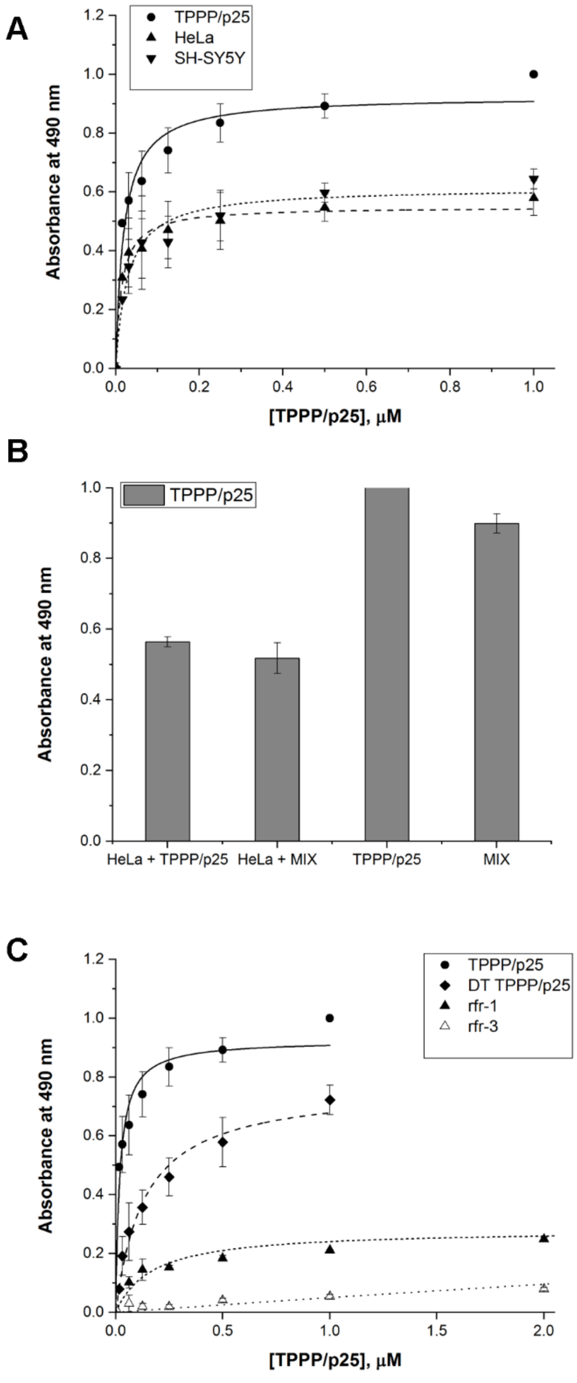

Supplementary Figure 2. Interaction of LC3B with TPPP/p25 as detected by ELISA. (A) Binding of human recombinant TPPP/p25 (●) on its own and added to HeLa (▲) or SH-SY5Y (▼) cell extracts to the immobilized LC3B. The initial concentration of TPPP/p25 in the 1 mg/ml cell extracts was 1 µM which was diluted, and the bound TPPP/p25 was quantified by specific anti-TPPP/p25 as described in the Material and Methods. The binding affinities evaluated by curve fitting, assuming simple hyperbolic saturation, are as follows: Kd (TPPP/p25) = 19.2 ± 4.4 nM (n=4), Kd (HeLa) = 14.1 ± 2.6 nM (n=3) and Kd (SH-SY5Y) = 28.4 ± 5.9 nM (n=3), respectively. (B) Interaction of

LC3B with TPPP/p25 and TPPP/p25 complexed with SYN in the absence and the presence of HeLa cell-free extract. Experiment conditions are similar as described for the ELISA experiment presented in (A) with the exception that the concentrations of proteins (1 μM) in the absence and the presence of the Hela extract (0.5 mg/ml) were constant. (C) Binding of deletion mutants of TPPP/p25 with one or two LC3-interacting region motifs to the immobilized LC3B. The binding affinities evaluated by curve fitting, assuming simple hyperbolic saturation, are as follows: Kd (TPPP/p25) = 19.2 ± 4.4 nM (n=4), Kd (DT or Δ3-43/Δ175-219 TPPP/p25) = 133 ± 24 nM (n=3) and Kd (rfr-1 or 43-90 TPPP/p25) = 167 ± 57 nM (n=4), respectively. rfr-3 or 142-219/Δ178-187 TPPP/p25, n=4.

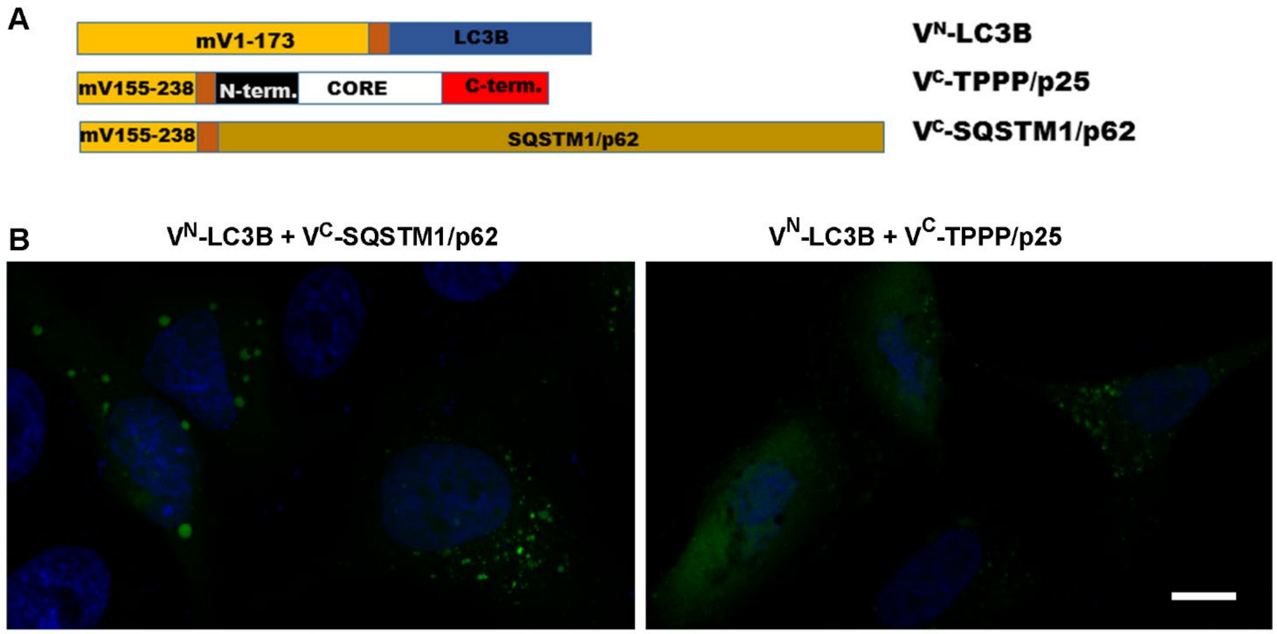

Supplementary Figure 3. Association of TPPP/p25 with LC3B in HeLa cells as visualized by BiFC technology. (A) Scheme of BiFC constructs for generation of BiFC signal (green) by co-transfection of V^N-LC3B and V^C-TPPP/p25 or V^C-SQSTM1/p62 fused into splitted mVenus BiFC constructs. (B) BiFC signal of the V^N-LC3B and V^C-SQSTM1/p62 or V^C-TPPP/p25, respectively. Nuclei were counterstained with DAPI (blue). Scale bars: 10 μm.

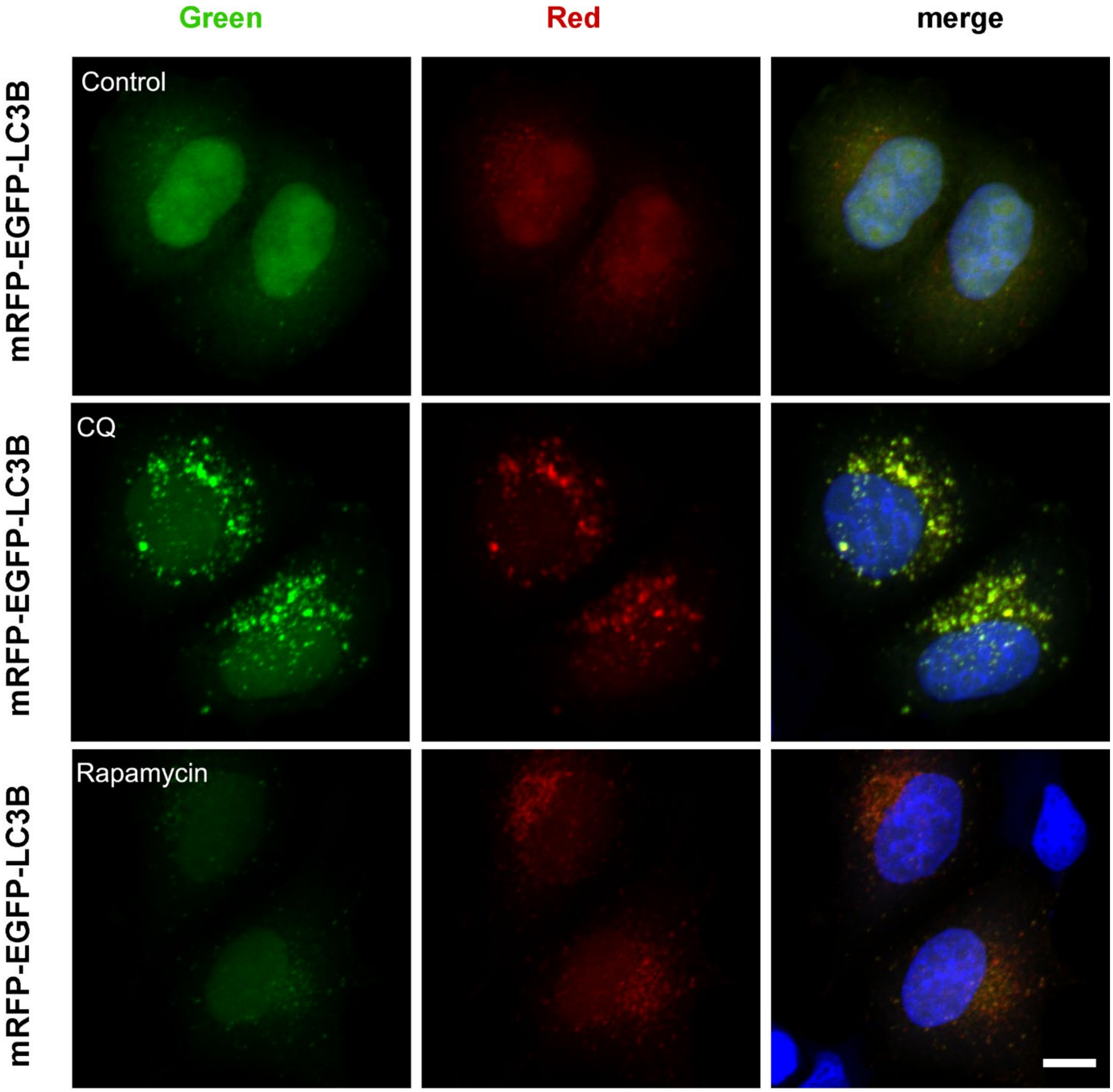

Supplementary Figure 4. Autophagy progression in control, CQ and rapamycin-treated LC3B-HeLa cells expressing tandem fluorescent-tagged LC3B (mRFP-EGFP) monitored by immunofluorescence confocal microscopy. The cells were treated with 5 μM CQ or 1 μg/ml rapamycin, when indicated. Nuclei were counterstained with DAPI (blue). Scale bar: 2.5 μm.

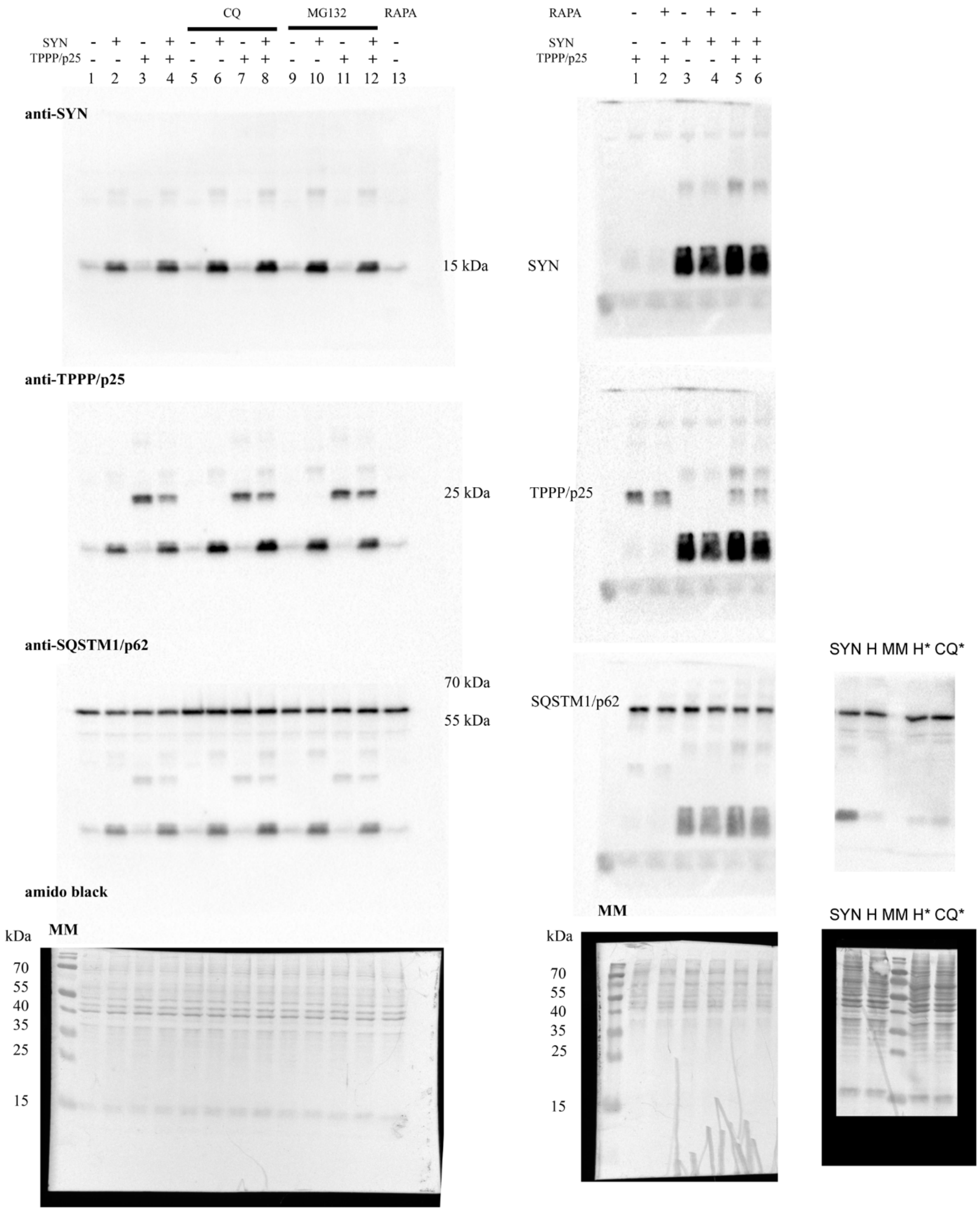

Supplementary Figure 5. Representative Western blot for the quantification of SYN, TPPP/p25 and SQSTM1/p62 levels in HeLa cells in the absence and presence of autophagy modulators. Samples: HeLa cells without (H*) and with (H) pre-starvation. The concentration of CQ, rapamycin (RAPA) and MG132 was 5 μM, 1 μg/ml and 5 μM, respectively. MM: molecular weight marker.

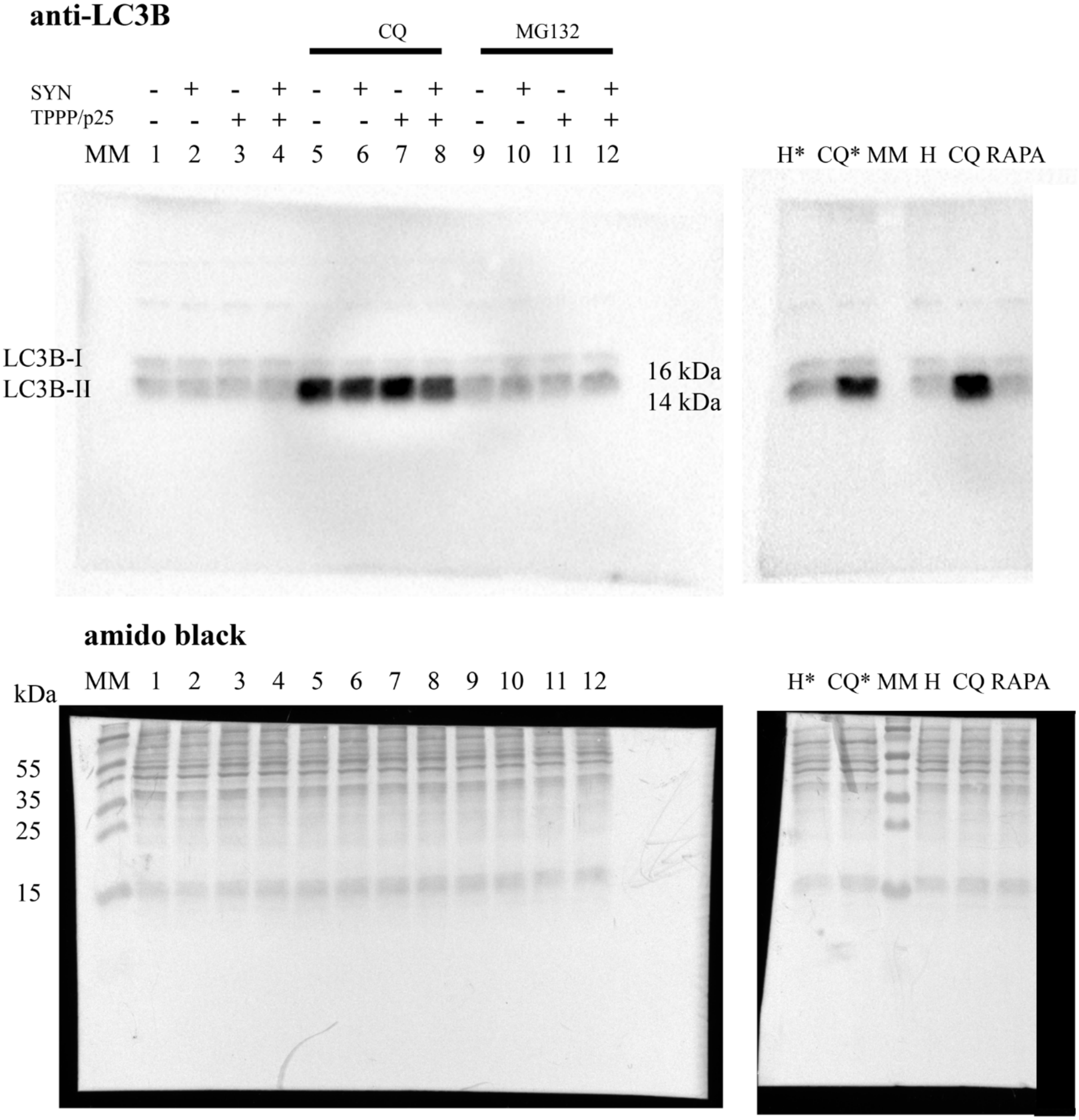

Supplementary Figure 6. Western blot for quantification of the LC3B-II levels in HeLa cells in the absence and presence of autophagy modulators. Samples: HeLa cells without (H*) and with (H) pre-starvation. The concentration of CQ, rapamycin (RAPA) and MG132 was 5 µM, 1 µg/ml and 5 µM, respectively. MM: molecular weight marker.

2.2 Supplementary Tables

Supplementary Table 1. The effect of small molecule modifiers on the LC3B-II and SQSTM1/p62 levels.

sample	LC3B-II level (average ± SD)	SQSTM1/p62 level (average ± SD)
starved cells	0.11 ± 0.08	1.00 ± 0.03
+CQ	1.00 ± 0.04	1.21 ± 0.20
+MG132	0.25 ± 0.08	1.03 ± 0.17
+Rapamycin	0.21 ± 0.07	0.89 ± 0.07
normal cells	0.23 ± 0.11	1.03 ± 0.07
+CQ	0.73 ± 0.14	1.22 ± 0.06

Statistical comparisons were performed with one-way ANOVA followed by Tukey's test. LC3B-II level: *** $p < 0.001$ for starved cells+CQ versus normal cells+CQ; **** $p < 0.0001$ for starved cells versus starved cells+CQ and normal cells versus normal cells+CQ. SQSTM1/p62 level: ** $p < 0.01$ for starved cells versus starved cells+CQ.
